# Myeloid- and epithelial-derived RELMα contribute to tissue repair following lung helminth infection

**DOI:** 10.3389/fpara.2023.1242866

**Published:** 2023-10-13

**Authors:** Stefanie N. Sveiven, Sang Yong Kim, Valeria Barrientos, Jiang Li, Jennell Jennett, Samuel Asiedu, Kyle Anesko, Tara M. Nordgren, Meera G. Nair

**Affiliations:** ^1^ Department of Biomedical Sciences, School of Medicine, University of California Riverside, Riverside, CA, United States; ^2^ Department of Environmental and Radiological Health Sciences, Colorado State University, Fort Collins, CO, United States

**Keywords:** RELMα, myeloid, epithelial cell, helminth, lung repair

## Abstract

Soil-transmitted helminth (STH) infections impact billions of individuals globally; however, there is a need to clarify the long-term impacts of these infections on pulmonary health owing to their transient migration and subsequent damage to the lungs. In mouse models of these infections using *Nippostrongylus brasiliensis*, lung pathology persists at later time points post single infection. These studies also indicate the persistent transcriptional expression of resistin-like molecule α (RELMα), an immunomodulatory protein induced in type 2 immunity and alternatively activated macrophages. Using constitutive and tamoxifen-inducible cell-specific RELMα knockout mouse strains, we identified that epithelial- and myeloid-derived RELMα protein remained elevated at 30 days post infection and altered the immune cell signature and gene expression in lung compartments. Histopathological assessment of alveolar damage revealed a role for RELMα in tissue repair, suggesting the importance of sustained RELMα expression for lung recovery from helminth infection. Acellular three-dimensional (3D) lung scaffolds were prepared from the lungs of wild-type (WT), RELMα KO-naive, or 30 days post *N. brasiliensis*-infected mice to assess their ability to support epithelial cell growth. *N. brasiliensis* infection significantly altered the scaffold and impaired epithelial cell growth and metabolic activity, especially in the RELMα KO scaffolds. These findings underscore a need to identify the long-term impacts of helminth infection on human pulmonary disease, particularly as alveolar destruction can develop into chronic obstructive pulmonary disease (COPD), which remains among the top global causes of death. Translation of these findings to human protein resistin, with sequence homology to RELMα therapeutic opportunities in lung repair.

## 1 Introduction

Soil-transmitted helminth (STH) infections impact 24% of the world’s population, particularly among the poorest and most vulnerable communities, and are considered a neglected tropical disease (NTD) (World Health Organization, 2023). The host life cycle of these STH infections begins with the infection of larvae in feces-contaminated soil; the larvae of human hookworms *Necator americanus* and *Ancylostoma duodenale* then migrate through the lung to be coughed up and swallowed into the gastrointestinal tract (Loukas et al., 2016). The transient migration of these hookworms through the lung results in chemical and mechanical injury to the vasculature and pulmonary epithelium, the long-term implications of which are not well understood in human infection. However, mouse models of these infections, using the hookworm *Nippostrongylus brasiliensis*, have indicated progressive long-term lung pathology, resembling emphysema, following a single infection with *N. brasiliensis* (Marsland et al., 2008; Reece et al., 2008; Heitmann et al., 2012; Bouchery et al., 2017).

*N. brasiliensis* transiently migrates through the lungs 2–4 days post infection. We and others have reported infection-induced hemorrhaging in the lungs, eosinophilia, alveolar destruction, and upregulation of the immunomodulatory protein resistin-like molecule α (RELMα) during these early time points, which may parallel the lung damage in human helminth infection (Reece et al., 2006; Pine et al., 2018; Kim et al., 2022; Houlder et al., 2023). The host immune response to helminth parasites is dominated by T helper type 2 (Th2) cytokines, airway eosinophilia, and macrophage recruitment. These responses mediate parasite killing and promote tissue repair (Chen et al., 2014; Nair and Herbert, 2016; Krljanac et al., 2019). RELMα (*Retnla)* is a secreted protein induced by Th2 cytokines and is upregulated following *N. brasiliensis* infection in the airway, as measured by bronchoalveolar lavage (BAL) and systemically in the serum within the first week of infection (Chen et al., 2016). Studies examining the progressive development of emphysema post infection also demonstrate continuous expression of RELMα transcripts at these time points (Marsland et al., 2008). A role for RELMα in tissue repair (promoting angiogenesis, proliferation, fibroblast differentiation, and collagen cross-linking) has also been reported, although the function of RELMα during the later post-infection phases needs further clarification (Knipper et al., 2015). The cellular source of RELMα also differs depending on the infection and tissue-type (Pine et al., 2018). RELMα is recognized as a hallmark protein of alternatively activated macrophages, or M2 macrophages, induced by the Th2 cytokine interleukin 4 (IL-4); however, this nomenclature is oversimplified given the recognized heterogeneity of macrophages (Murray et al., 2014; Kim and Nair, 2019). Although RELMα expression is elevated in all myeloid cells following helminth infection or Th2 cytokine treatment, the highest level of RELMα expression is reported in monocyte-derived macrophages and not embryonically seeded macrophages (Guilliams et al., 2013; Svedberg et al., 2019; Li et al., 2021; Sanin et al., 2022). RELMα also plays a homeostatic role in myeloid homeostasis; RELMα deficiency in myeloid progenitors leads to a decrease in monocyte-derived macrophages in the tissue (Sanin et al., 2022). Airway epithelial cells also express RELMα in helminth infection and allergic inflammation (Holcomb et al., 2000; Pine et al., 2018); however, the functional impact of epithelial-cell-derived RELMα has not been explored in chronic inflammation induced by lung helminth infection.

Based on these previous studies, the focus of this project was to elucidate the cellular source of persistently elevated RELMα and the impact on emphysematous lung pathology using three RELMα-targeted gene knockout mouse models: a whole-body RELMα knockout system and CD11c- and CC10-cell-specific Cre recombinase knockout systems, which target alveolar macrophage (AM) and lung epithelial cell contributors of RELMα, respectively. For these transgenic mouse systems, the CD11c–Cre strategy may also target dendritic cells and some lymphocyte subsets, whereas the tamoxifen-inducible CC10–Cre system would target the club cells in the lung at the time of tamoxifen treatment (Caton et al., 2007; Rawlins et al., 2009; Guilliams et al., 2013; Abram et al., 2014). At day 30 post single infection with *N. brasiliensis*, we analyzed the RELMα-dependent responses from peripheral and resident immune cell populations with the goal of identifying if RELMα is tissue protective in progressive helminth-induced alveolar destruction. We also employed an *ex vivo* repair model using acellular scaffolds from infected and naive lungs of both wild-type (WT) and knockout (KO) mice to assess if RELMα alters epithelial repair via the extracellular matrix (ECM). These studies revealed cell-intrinsic effects of RELMα expression by peripheral and resident macrophage populations, as well as impacts on lung histopathological outcomes and ECM–epithelial interactions.

## 2 Materials and methods

### 2.1 Transgenic mice

The *Retnla*TdT (RELMα^KO^) mice were generated by genOway, as described previously (Li et al., 2021). These mice were backcrossed to C57BL/6J to generate KO and WT controls, bred in-house. The *Retnla*-floxed mice were also generated by genOway in a similar way to the *Retnla*TdT mice, and bred in-house to generate the *Retnla* ^F/F^ mice. The B6.Cg-Tg(Itgax-Cre)1-1Reiz/J (strain #: 008068; common name: CD11c-Cre) and B6N.129S6(Cg)-Scgb1a1tm1(Cre/ERT)Blh/J (strain #: 016225; common name: Scgb1a1-CreER™ or CC10-Cre) mice were purchased from the Jackson Laboratory. The Cre heterozygous CD11c-Cre and CC10-Cre mice were bred in-house with *Retnla* ^F/F^ mice to generate RELMα^ΔCC10^, RELMα^ΔCD11c^, and corresponding F/F controls. The RELMα^ΔCC10^ mice are tamoxifen-inducible and were administered five 100-µL doses of tamoxifen (Sigma) in corn oil at a concentration of 20mg/mL intraperitoneally every other day. Following the fifth dose, the mice were given 7 days to recover prior to helminth infection. The mice were sex and age matched (6–13 weeks) and housed in a specific pathogen-free vivarium with standard 12-hour light cycles. The mice had *ad libitum* access to standard chow and water. The animals were anesthetized with 2% volume-to-volume (v/v) isoflurane using a vaporizer approved for small animals, with a negative hind pedal reflex indicating sufficient anesthesia. Isoflurane was used for humane euthanasia followed by exsanguination for tissue harvest. The animal experiments were conducted in accordance with National Institutes of Health guidelines, the Animal Welfare Act, and the Public Health Service Policy on Humane Care and Use of Laboratory Animals. The protocols for the use of these animals were approved by and in accordance with the guidelines of the University of California Riverside Institutional Animal Care and Use Committee (IACUC, protocol number A-20210017).

### 2.2 *Nippostrongylus brasiliensis* infection

The mice were infected subcutaneously with L3 larvae from *N. brasiliensis*, amounting to 600 worms in 200 µL of saline, as described previously (Batugedara et al., 2018; Li et al., 2021). The mice were given 30 days to recover from this single infection and were euthanized on day 30 following infection on day 0. For the acellular scaffold studies, mice were euthanized on day 80 post infection. Weight was monitored to prevent egregious weight loss in these animals in the few days after the initial infection.

### 2.3 Tissue harvest

After euthanasia, blood was collected from the renal arteries; the blood was allowed to coagulate and was then processed for serum by centrifugation. A small incision was made, revealing the trachea, to fit the cannula, a luer-lock shield from an 18-gauge shielded intravenous (IV) catheter (cat # 381447; BD Insyte™ Autoguard™). This was tied to the trachea with a nylon suture and used with a 1-mL syringe to perform the BAL using 2 × 800 µL of cold phosphate-buffered saline (PBS). The supernatant from the first BAL wash was used for protein analyses, whereas the cell pellets were used for flow cytometry. Prior to the excision of the heart and lungs en bloc, vascular immune cells were cleared from the lungs by cardiac perfusion with PBS. A nylon suture was tied around the right main bronchus, and the right lung lobes were removed and processed to create a single-cell suspension for flow cytometry and RNA analysis. The excised right lobes were minced using a scalpel and then incubated in a digestion solution [containing 5% fetal bovine serum (FBS), 1 mg/mL of Roche’s collagenase A, and 0.05 mg/mL of Sigma’s DNase I in Hank’s balanced salt solution (HBSS)] for 1 hour while being shaken at 37.5°C. At 15 minutes prior to the final 1-hour mark, the lungs were homogenized through a 18-gauge needle (10 times) and returned for the final 15 minutes of 37.5°C incubation. The incubated sample was poured over 70-µm cell strainers and pelleted. Red blood cell (RBC) lysis was performed in accordance with the manufacturer’s protocol [BioLegend (10X)]. The cells were resuspended in PBS and cell counts were quantified using an automated cell counter (DeNovix CellDrop™). Up to 1 million cells of each sample were used for RNA extraction and gene expression analysis by NanoString. Where indicated, lung suspensions were stained with αCD11c for sorting of CD11c^+^ cells on the MoFlo Astrios cell sorter (Beckman Coulter) prior to NanoString analysis, as previously described (Batugedara et al., 2018).

### 2.4 Histology and immunofluorescence

The lung lobes were prepared for either formalin-fixed paraffin embedding (FFPE) or cryopreservation. For FFPE, the left lobe was partially inflated with 300 ML of 10% buffered formalin via the tied cannula and placed on an apparatus used to inflate the lungs under a constant pressure. This apparatus uses the pressure of 10% buffered formalin at 22 cm above the lungs for inflation, with the lungs surrounded by 10% buffered formalin, using reagent reservoirs; the lungs were sent to the Histology Core Facility at Sanford Burnham Prebys or the Translational Pathology Core Laboratory at UCLA for paraffin embedding. Hematoxylin and eosin (H&E) staining was also performed using these histology cores on 5-µm FFPE lung sections. For optimal cutting temperature (OCT) embedding, lungs were inflated with two parts OCT compound and one part formalin warmed to 60°C. The lungs were submerged in 30% sucrose formalin overnight, then in 30% sucrose PBS overnight. The lungs were embedded in OCT compound over dry ice and sectioned by using a cryotome at 5 µm. The sections were stored at −20°C until they were used for immunofluorescent staining. After washing slides in PBS to remove the OCT compound, slides were blocked for 1 hour (StartingBlock™ Blocking Buffer, cat. 37542; Thermo Scientific™). The slides were labeled with anti-murine CC10 (1: 200, clone: B-6; Santa Cruz Biotechnology), biotin-conjugated anti-murine RELMα (1: 200, cat. 500-P214; PeproTech®), in blocking buffer overnight at 4°C. The slides were washed three times in PBS with 0.05% Tween-20, then labeled with secondary antibody [goat anti-rabbit IgG fluorescein isothiocyanate (FITC), streptavidin-conjugated Cy5, 1: 300] for 2 hours at room temperature, washed three times, then coverslipped with mounting medium containing 4′,6-diamidino-2-phenylindole (DAPI; VECTASHIELD® antifade mounting medium with DAPI, cat. H-1200-10; Vector Laboratories).

### 2.5 Flow cytometry

The BAL cells were pelleted by centrifugation at 400 g for 5 minutes, then RBCs were lysed. The cells were resuspended in PBS and cell counts were quantified using an automated cell counter (DeNovix CellDrop™). For flow cytometry, the cells were pelleted in v-shaped-bottom 96-well plates. The panel of BioLegend antibodies used for flow analysis was as follows, used at 1: 400: FITC anti-mouse MERTK (Mer) antibody (clone: 2B10C42; BioLegend); Alexa Fluor® 700 anti-mouse major histocompatibility complex class II (MHCII; I-A/I-E) antibody (clone: M5/114.15.2; BioLegend); allophycocyanin (APC)/cyanine 7 (Cy7) anti-mouse CD11b antibody (clone: M1/70, BioLegend); Brilliant Violet 510™ anti-mouse Ly-6G antibody (clone 1A8; BioLegend); Zombie Aqua™ Fixable Viability Kit, Brilliant Violet 605™ anti-mouse CD11c antibody (clone: N418; BioLegend); Brilliant Violet 650™ anti-mouse F4/80 antibody (clone: BM8; Biolegend); phycoerythrin (PE)-CF594 rat anti-mouse Siglec-F (clone: E50-2440; BD Biosciences); and PE/Cy7 anti-mouse Ly-6C antibody (clone: Hk1.4; BioLegend). The samples were acquired using the NovoCyte Quanteon flow cytometer (Agilent Technologies) and flow data were analyzed using FlowJo™ version 10.

### 2.6 Enzyme-linked immunosorbent assay

Recombinant murine RELMα protein, rabbit-anti-murine RELMα, and biotinylated-rabbit-anti-murine RELMα were purchased from PeproTech (cat # 450-26, 500-P214, and 500-P214BT, respectively). The plates were coated overnight at 4°C with rabbit anti-murine RELMα at a concentration of 0.5 µg/mL, and the ELISA was completed the following day. A detection antibody was also used at a concentration of 0.5 µg/mL.

### 2.7 Histopathology assessment

The H&E-stained slides were blinded and scored from 1 to 5 on alveolar destruction and vascular inflammation across the lung, which was divided into five equally spaced sections from the apex to the base. Each of the five spaces was assessed for the percentage of alveoli damaged and scored. The scores were reported as an average for each mouse. The scoring parameters were as follows: 0 for no detectable damage, 0.5 for 5% damaged, 1.0 for 10% damaged, 1.5 for 20% damaged, 2.0 for 30% damaged, 2.5 for 40% damaged, 3.0 for 50% damaged, 3.5 for 60% damaged, 4.0 for 70% damaged, 4.5 for 80% damaged, and 5 for ≥90% damaged.

### 2.8 Worm binding assay

The L3 *N. brasiliensis* larvae were isolated from fecal plates and treated with antibiotics prior to being placed in a sterile culture [containing 0.012 g of neomycin (cat. N1876; Sigma Aldrich), 1% penicillin–streptomycin (cat. 15140122; Fisher Scientific), and 30 mL of sterile PBS]. The lungs from RELMα KO and WT mice were collected on day 9 post infection with *N. brasiliensis*. The lungs were then digested, as mentioned above, and single-cell suspensions of lung homogenate were enriched for CD11c^+^ cells for use in the worm coculture experiments. Enrichment was performed by anti-CD11c magnetic bead isolation methods in accordance with the manufacturer’s protocol (cat. 130–125–835; Miltenyi Biotec). Flow cytometry and cell appearance on cytospin analysis were used for confirmation of CD11c+ cell enrichment. Then, 400,000 CD11c-enriched cells and 25 live L3 larvae were added to each well of a 24-well plate. The culture medium [500 µL per well; 10% FBS, 1% penicillin–streptomycin, 1 mM sodium pyruvate, 25 mM HEPES in Dulbecco’s modified Eagle’s medium (DMEM)] was also supplemented with serum collected from memory-infected mice deficient in RELMα. Cocultures were treated with an isotype control (cat. I413; Sigma Aldrich; rat IgG 100 microg/mL), anti-murine CD11a (cat. 101101; BioLegend; 2.5 µg/mL), or anti-murine integrin β7 (cat. 321202; BioLegend; 5 µg/mL) at the time of plating. The images were captured 24 hours after plating and cells bound to individual worms were counted from the images.

### 2.9 NanoString

CD11c+ myeloid cells were sorted from lung cells at day 7 post infection (WT or RELMα KO), lysed, and directly hybridized to the mouse Myeloid Innate Immunity panel (cat. XT-CSO-MMII2-12; NanoString). The log2-transformed values were obtained after positive and negative normalization, and calculated as the geometric mean of corresponding values. Differential expression analysis was conducted with *p*-values identifying significantly altered genes at a *p*-value < 0.05 as indicated. Gene set analysis (GSA) was conducted using NanoString advanced analysis algorithms for the Myeloid Innate Immunity panel, facilitated by the ROSALIND® platform. The GSA scores were determined by averaging significance measures across genes within each pathway. Significance scores quantified overall pathway changes and were derived by amalgamating differential expression *t*-tests for all genes within a pathway.

For the lung cells, RNA was isolated from single-cell suspensions of lung homogenate 30 days post infection (WT/RELMα^ΔCC10^/RELMα KO), in accordance with the manufacturer protocol (cat. Z3101, SV Total RNA Isolation System; Promega). Then, 50 ng of total RNA was hybridized to the mouse immunology panel (cat. XT-CSO-MIM1–12; NanoString). The NanoString gene expression data were processed and analyzed using the NanoTube package in R (The R Foundation for Statistical Computing, Vienna, Austria). The genes were filtered based on adjusted *p*-values (*p*_adj_) with a threshold of < 0.05, identifying differentially expressed genes between experimental groups. The Qiagen Ingenuity Pathway Analysis (IPA) tool was employed to conduct the gene pathway analysis. Relevant differentially expressed pathways with their represented percentage of genes were identified through IPA analysis.

### 2.10 Acellular scaffold assay

The mice were euthanized on day 80 post infection and perfused by cardiac perfusion with PBS until they turned white. Acellular scaffolds were prepared using an adapted protocol for the decellularization of human lungs from our previous publication (Nordgren et al., 2018). A cannula was placed in the trachea, as in the BAL washes above, and tied tightly with nylon suture wire. The lungs were gently excised, inflated with 1 mL of 0.1% Triton X in deionized, distilled water (ddH_2_O), and the syringe was left attached to maintain the volume in the lungs. The lungs were then placed in a plastic container and covered with ddH_2_O for overnight incubation at 4°C. The next day, the lungs were deflated and reinflated with 2% deoxycholic acid (Spectrum) and placed back in the container with fresh ddH_2_O for overnight incubation at 4°C. On the final day, the lungs were deflated and rinsed with 2% p/s PBS (inflated deflated). Prior to vibratome sectioning, the lungs were inflated with 2% low-melting-point agarose in PBS and chilled on ice to set the agarose. The lungs were sectioned with the manufacturer’s 300-µm-thick tungsten carbide blades and placed in 100% ethanol for sterilization and cold storage (Precisionary Instruments, Compresstome®; speed setting: 1, oscillation setting: 10). To standardize lung size, sections were identified by cut number and only cuts 6–10 were used in these studies. Hoechst staining of representative scaffolds revealed successful decellularization. Prior to the assay, scaffolds were incubated in 2% penicillin–streptomycin in PBS overnight at 37.5°C to clear the agarose. The following day, the 2% p/s in PBS was replaced for two more 20-minute washes at 37.5°C. The murine lung epithelial cell line MLE 12-CRL2110™ [American Type Culture Collection (ATCC®), passages 6–8] was plated at 200,000 cells per scaffold in 48-well plates to assay for *ex vivo* lung repair. The cells were plated directly onto the scaffolds in 100 µL of ATCC-recommended HITES medium for overnight incubation at 37.5°C in a humidified cell culture incubator at 5% CO_2_. The following day, an additional 200 µL of HITES medium was added to each scaffold. On day 3, the medium was replaced. On day 6, the scaffolds were stained with Hoechst dye (1: 2,500 of 20 mM, cat #) and transferred to a new 24-well plate for imaging with a 4X objective using the Keyence BZ-X810 fluorescence microscope. After imaging, scaffolds were gently transferred to new 24-well opaque plates for analysis of cellular ATP, in accordance with the manufacturer’s protocol (cat # G9681, CellTiter-Glo™ 3D Cell Viability Assay; Promega). This allowed for the quantification of ATP from only those cells associated with the scaffold. The protein content of each scaffold was determined by bicinchoninic acid assay (Pierce™ BCA kit). Cellular ATP was normalized to the respective scaffold protein content to adjust for differences in available surface for cell adherence. A nuclei count analysis was performed using an automated protocol from the University of Chicago (Labno, 2014).

### 2.11 Statistics

Statistical analyses were performed using GraphPad Software Prism 9 (GraphPad Software Inc., CA, USA) and R software (version 2023). With the exception of the NanoString analyses, which were performed on one experiment only (CD11c^+^ cells), and specimens from pooled experiments (lung cells), the experiments were repeated at least twice with a number (*n*) of three or more per group. Outliers were removed by ROUT. The group analyses were performed via two-way ANOVA followed by Tukey’s *post hoc* multiple comparisons analyses and plotted as bar graphs showing the mean with the standard error. *In vitro* analyses were averaged per group per experiment and analyzed by one-way ANOVA or *t*-test. These were graphed as box and whisker plots. All graphs were formatted using the “colorblind safe 4” color palette in Prism.

## 3 Results

### 3.1 Cell-specific deletion of RELMα from myeloid cells versus epithelial cells differentially impacts RELMα protein secretion in the alveolar spaces and the blood.

**Figure 1:**
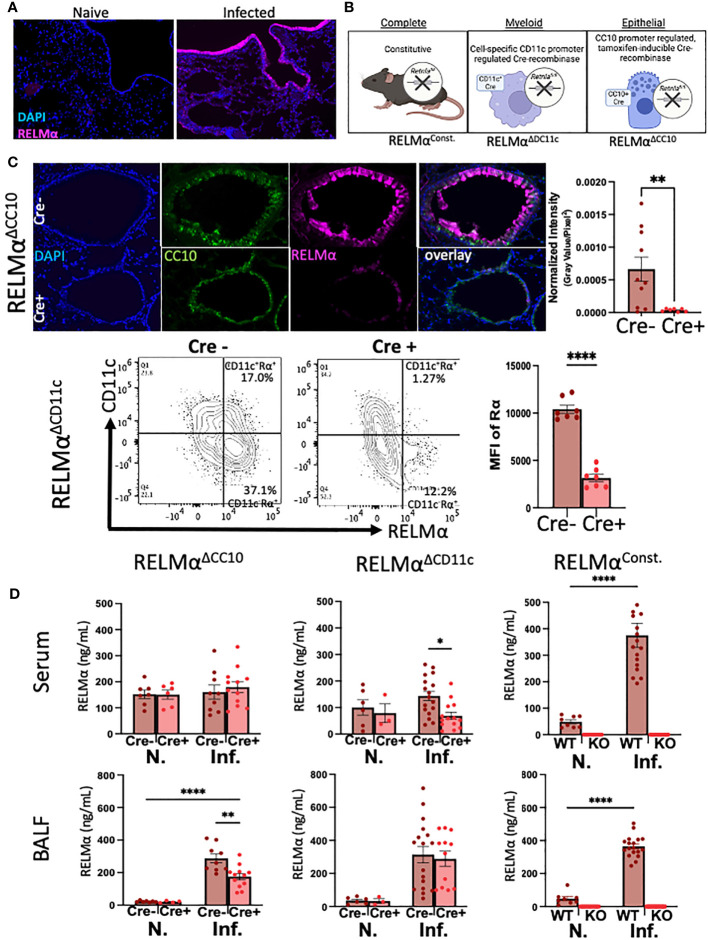
Cellular sources of RELMα drive compartment-specific expression. **(A)** Immunofluorescence (IF) on day 7 reveals expression of RELMα by airway epithelium and mesenchymal cells (OCT, 5 µm). **(B)** Schematic of mouse strains for constitutive and cell-specific knockout. **(C)** Validation of cell-specific deletion of RELMα by IF and intracellular flow staining shows a significant reduction in RELMα intensity in the airways (RELMα^ΔCC10^) and CD11c^+^ cells (RELMα^ΔCD11c^) at day 7 post infection. **(D)** Cell-specific deletion of RELMα reveals elevated levels of expression in bronchoalveolar lavage owing to CC10^+^ epithelial cells at day 30 post infection, whereas serum RELMα is maintained by CD11c^+^ myeloid cells. Experiments were performed in duplicate or triplicate. Individual animals are plotted as means with standard errors and analyzed by two-way ANOVA with Tukey’s *post hoc* comparisons. *p < 0.05, **p < 0.01 and ****p < 0.0001.

RELMα was expressed by macrophages and lung epithelial cells at day 7 post infection, as shown by immunofluorescent staining in the airway and mesenchyme of lung sections (**Figure 1A**). We validated the effectiveness of our complete, myeloid (CD11c-Cre)-, and epithelial (CC10-Cre)-specific RELMα knockout systems **(** **Figure 1B** **)** using a variety of methods (e.g., immunofluorescence, intracellular flow cytometry, and ELISA). Because flow cytometry of epithelial cells was challenging owing to the low viability of these cell subsets, RELMα^ΔCC10^ mice were validated by immunofluorescent staining of the airways with fluorescent quantification, revealing a significant reduction in RELMα fluorescent intensity in infected Cre^+/–^ mice (RELMα^ΔCC10^) at day 7 compared with Cre^–/–^ (RELMα^F/F^) mice (**Figure 1C**). RELMα^ΔCD11c^ mice were validated by intracellular flow cytometry, which demonstrated a reduction in the frequency of RELMα^+^CD11c^+^ cells and in the mean fluorescence intensity (MFI) for RELMα. The CD11c^+^ subset comprised interstitial macrophages and dendritic cells, which are also likely sources of RELMα, in addition to alveolar macrophages, which were higher in frequency than the other subsets (Cook et al., 2012; Svedberg et al., 2019). The cell-specific knockouts allowed us to clarify if epithelial or myeloid cells were responsible for maintaining RELMα levels in the peripheral and lung-specific compartments at day 30 post infection (**Figure 1D**). The RELMα^ΔCC10^ mice had reduced levels of RELMα in the bronchoalveolar lavage fluid (BALF), with levels unaffected in serum. Conversely, CD11c^+^ cells contributed to the serum RELMα levels, since RELMα^ΔCD11c^ mice exhibited a significant reduction in RELMα levels in the serum but not in the BALF. Thus, RELMα concentration at day 30 post infection was maintained by specific cell populations in a compartment-dependent manner, with lung-specific RELMα protein contributed by CC10^+^ lung epithelial cells, and systemic expression in the blood contributed by CD11c^+^ cells.

### 3.2 Distinct effects of whole-body versus cell-specific deletion of RELMα on peripheral and resident cell populations in the pulmonary compartment

**Figure 2:**
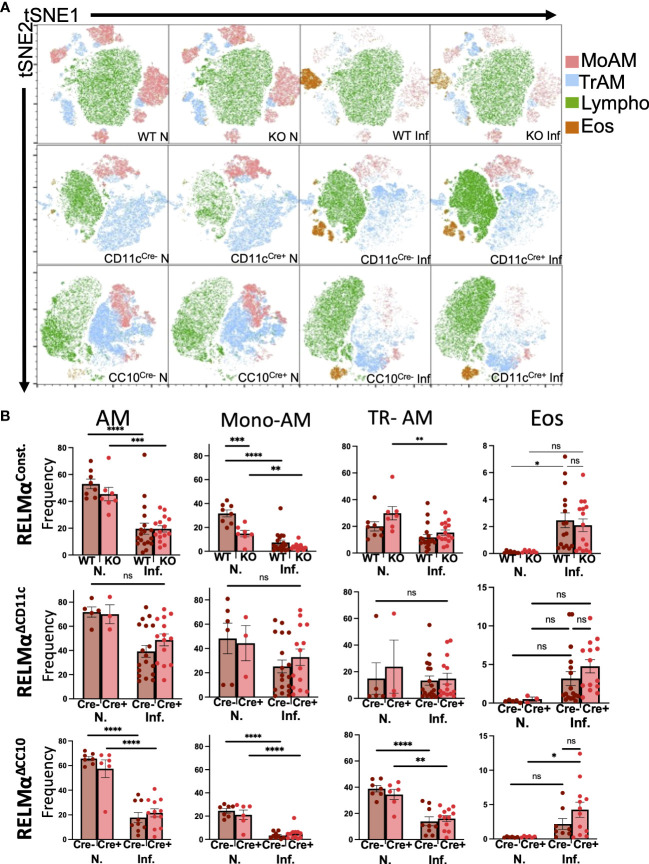
Alveolar macrophage populations in the airway are reduced at chronic time points post infection. **(A)** Uniform manifold approximation and projection (UMAP) analyses performed on representative experiments show infection-induced population changes in the bronchoalveolar lavage cells at day 30 post infection across all genotypes (RELMα^ΔCD11c^, RELMα^ΔCC10^, RELMα KO, and WT). **(B)** Alveolar macrophages (Siglec-F^+^ CD11c^+^), alveolar macrophage subsets (tissue-resident, TR; monocyte-derived, Mo), and eosinophil frequencies were determined in naive and infected mice at 20 days post infection. Individual animals are plotted as means with standard errors and analyzed by two-way ANOVA with Tukey’s *post hoc* comparisons. *p < 0.05, **p < 0.01, ***p < 0.001, and ****p < 0.0001, ns= not significant.

Lung infection or injury markedly alters macrophage populations, causing the recruitment of monocyte-derived alveolar macrophages (Mo-AMs) from the blood that replenish the tissue-resident AM (TR-AM) population, which are originally derived from the fetal liver and yolk sac (Guilliams et al., 2013; Misharin et al., 2017). Mo-AMs recruited following *N. brasiliensis* infection were also shown to express RELMα. The long-term effects of *N. brasiliensis*-induced lung inflammation and AM populations in the RELMα transgenic models were evaluated at day 30 post infection. Owing to the extended time point, intestinal worm counts could not be performed. Instead, infections of these mice were assessed by fecal egg burden (**Figure S1**). Quantification and flow cytometry of the BAL cells were performed at day 30 post infection in WT mice or mice where RELMα was constitutively deleted or deleted in CC10- or CD11c-expressing cells (**Figure S2**). AMs in the BALF were evaluated and grouped based on CD11c and Siglec-F co-expression, followed by MHCII expression; TR-AMs are gated as MHCII^lo^ while Mo-AMs are MHCII^HI^, based on previous studies (Li et al., 2022). Interstitial macrophages and dendritic cells are also MHC class II^HI^; however, they are not Siglec-F positive nor are they typically present in the BAL. Uniform manifold approximation and projection (UMAP) analysis was performed and revealed infection-induced eosinophils and decreased AM populations (**Figure 2A**). Quantification of AMs and eosinophil frequencies in naive and day 30 post infection samples across all genotypes was performed **(** **Figure 2B** **)**. Overall, AM frequencies, particularly that of Mo-AMs, were reduced with infection. Across all genotypes, eosinophil frequencies were increased with infection, even at this late time point. When comparing genotype-specific differences, no significant changes were observed with infection, in either constitutive or cell-specific RELMα KO mice. However, under naive conditions, Mo-AM frequency was significantly reduced in constitutive RELMα KO mice. In summary, there was an infection-dependent reduction in macrophage frequency, and an increase in eosinophil frequency in the BAL that persisted at the 30-day time point but was not RELMα dependent.

### 3.3 Both epithelial- and alveolar macrophage-derived RELMα are sufficient to promote lung tissue repair following helminth infection

**Figure 3:**
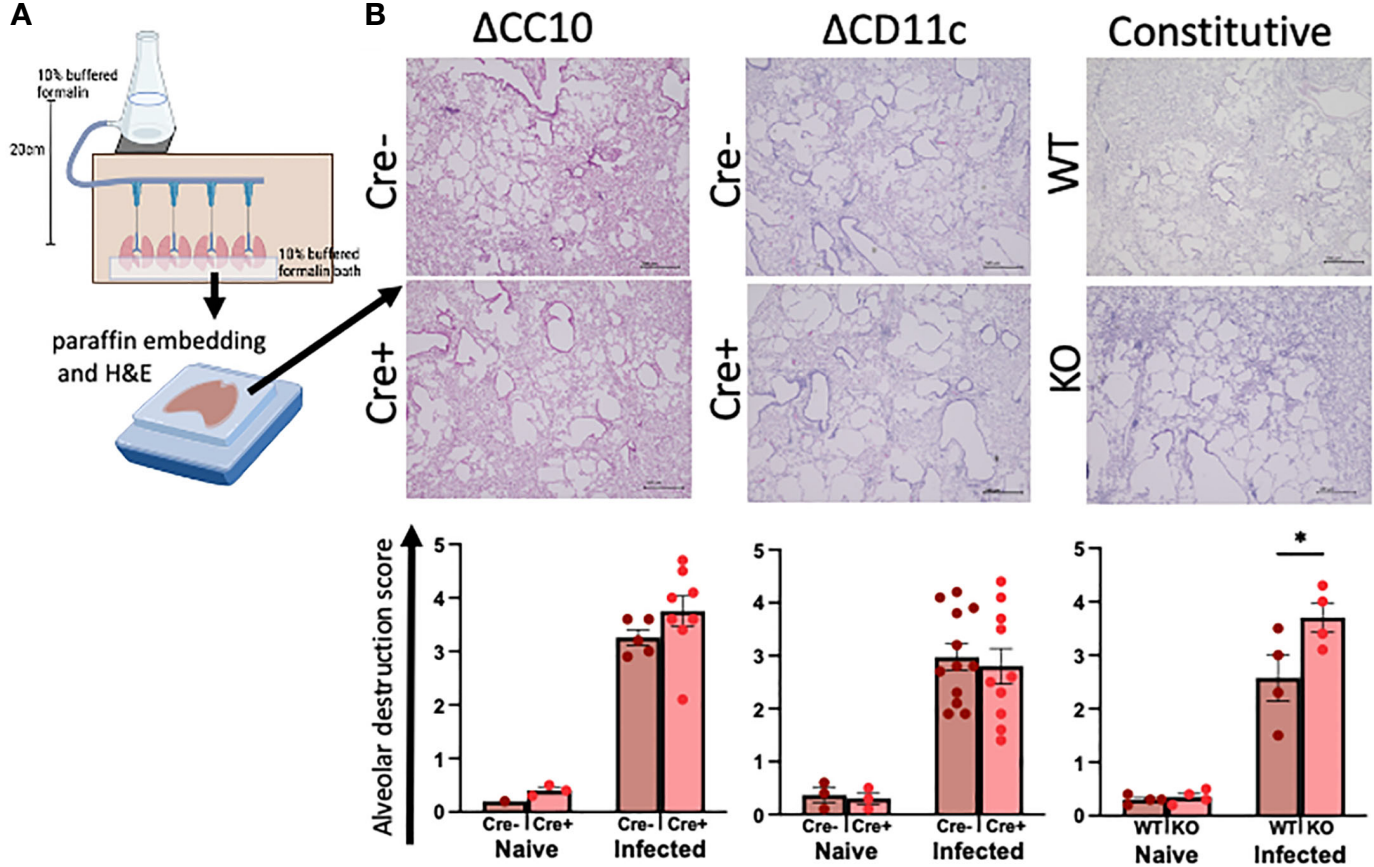
Deletion of RELMα exacerbates alveolar destruction pathology at day 30 post infection. **(A)** Schematic demonstrating the fixed-pressure apparatus used for formalin fixing of lungs prior to paraffin embedding (FFPE). **(B)** Representative images and alveolar destruction histopathology scores of alveolar spaces from hematoxylin and eosin (H&E)-stained FFPE sections (4× obj., 5 Mm). The slides were blinded prior to histopathology analyses. Individual animals are plotted as means with standard errors and analyzed by two-way ANOVA with Tukey’s *post hoc* comparisons. *p < 0.05.

The lungs were inflated with formalin at a constant pressure to ensure physiological airspaces for the comparison across mice of variable lung volumes prior to paraffin embedding (**Figure 3A**). Histopathological scoring of H&E-stained lungs was performed on blinded slides to assess the role of RELMα expression in alveolar destruction following helminth infection (**Figure 3B**). When compared with naive controls, infection resulted in higher scores for alveolar destruction regardless of RELMα status. Deletion of RELMα from either CC10^+^ or CD11c^+^ cells alone was insufficient to result in statistically significant changes; however, constitutive RELMα KO mice had increased levels of alveolar destruction at day 30 post infection, in line with previous studies conducted at earlier time points (Chen et al., 2016; Batugedara et al., 2018; Sutherland Id et al., 2018), which suggests a tissue-protective role for RELMα during helminth infection that can be derived from either epithelial cells or CD11c^+^ cells.

### 3.4 RELMα-deficient CD11c^+^ cells have dysregulated expression of genes related to migration and ECM remodeling and enhanced worm-binding through integrins

**Figure 4:**
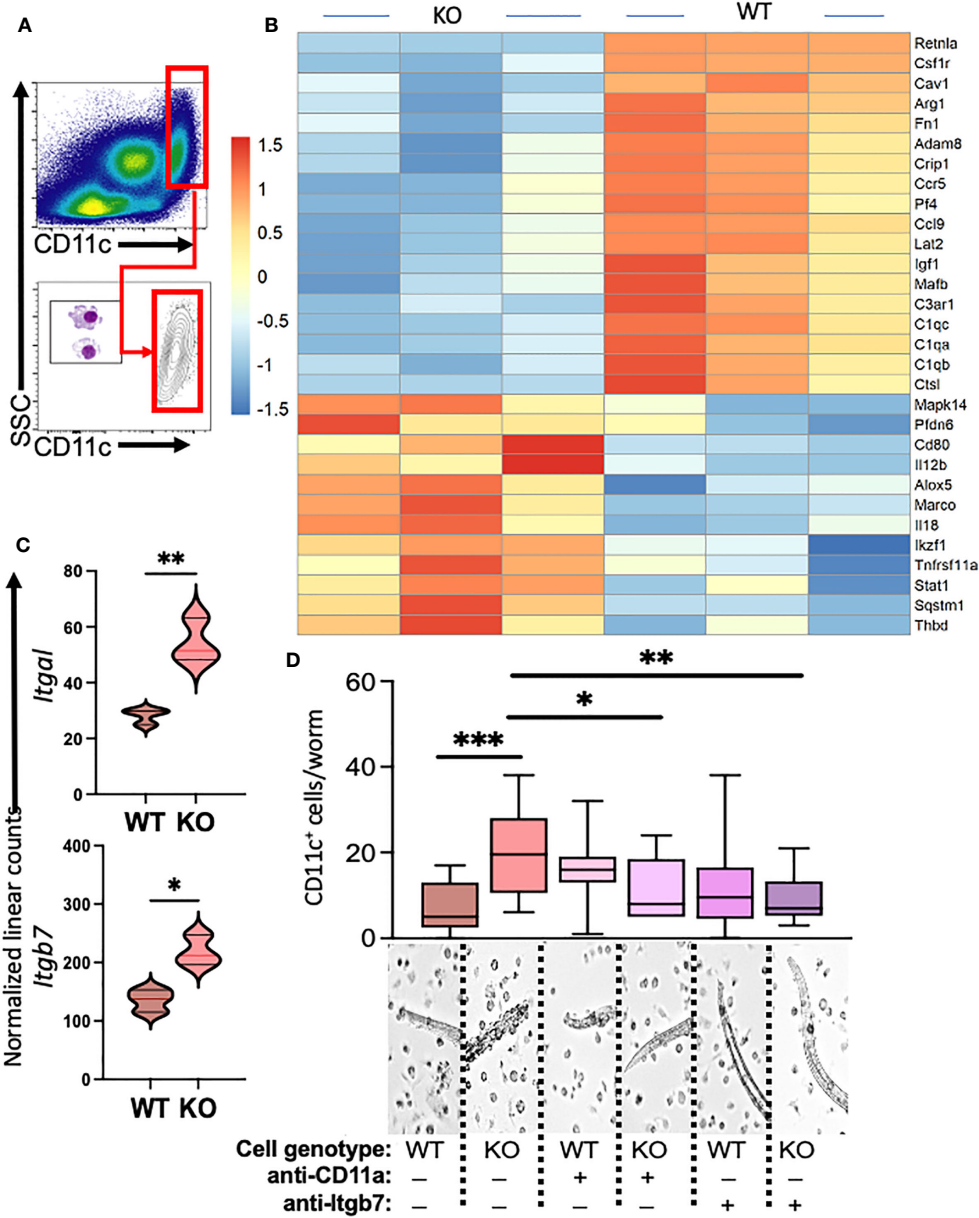
Myeloid cell-intrinsic RELMα expression at day 7 post infection alters transcriptional activation and host–pathogen interaction. **(A)** Cell-sorting analysis illustrating the isolation of CD11c^+^ cells from both WT and RELMα KO mice at 7 days post *Nippostrongylus brasiliensis* infection. Sorted CD11c^+^ cells were subsequently utilized for gene expression analysis with the NanoString Myeloid Innate Immunity panel. **(B)** Heatmap depicting the top 30 differentially expressed genes in CD11c^+^ cells isolated from infected WT and KO mice. **(C)** Normalized linear counts of *Itgal* and *Itgb7*. **(D)** Bead-enriched CD11c^+^ cells were cocultured with live *Nb* parasites and cell binding was quantified after 24 hours. Cells were either treated with isotype control or antibodies against CD11a (*Itgal*) or Itgb7. Box and whiskers depict the minimum and maximum; significance was calculated using single *t*-test comparisons of each experimental group with WT PBS as the control. *p < 0.05, **p < 0.01, ***p < 0.001.

Our previous investigations demonstrated distinctions in activity and function between lung myeloid cells from WT and those from constitutive RELMα KO mice (Batugedara et al., 2018). Notably, enhanced worm binding was observed by KO cells compared to WT cells. We aimed to extend our study into downstream genes responsible for enhanced worm binding that may also contribute to increased lung injury. CD11c^+^ cells were isolated from day 7 post infection lungs from WT and KO mice, and purity was confirmed by flow cytometry and cytospin analysis (**Figure 4A**). An additional anti-CD11c fluorescence minus one (FMO) control was performed and validated anti-CD11c specificity (**Figure S3**). The CD11c^+^ cell lysates were hybridized to the Myeloid Innate Immunity NanoString panel. Heatmap analysis of the top differentially expressed genes (DEGs) indicated that RELMα WT CD11c^+^ cells had increased levels of expression of genes associated with wound healing (*Fn1, Adam8, Arg1*, and complement protein *C1q*), while RELMα KO CD11c^+^ cells had increased levels of gene expression associated with Th1 cytokine-activated macrophages (*Cd80, Il12b*, and *Stat1*) (**Figure 4B**). Consistent with these findings, pathway analysis revealed that the DEGs mapped to functions associated with associated with cell migration, adhesion and ECM remodeling. RELMα KO CD11c^+^ cells exhibited Th1-skewed pathways, with increased significance scores for Th1 activation, toll-like receptor signaling, and T-cell activation (**Table 1**). This skewing toward inflammatory macrophage activation was at the expense of factors involved in the repair of the microenvironment needed to resolve alveolar damage and destruction. We identified two genes with increased levels of expression in RELMα KO CD11c^+^ cells that are involved in cell migration/adhesion: *Itgal* and *Itgb7* (**Figure 4C**) (Schittenhelm et al., 2017). We investigated these as potential effectors mediating the enhanced activation and binding of RELMα KO CD11c^+^ cells to the *N. brasiliensis* larvae. Blocking antibodies against *Itgal* and *Itgb7* were used in cocultures of *N. brasiliensis* larvae with WT and KO CD11c^+^ cells isolated from *N. brasiliensis*-infected mice. RELMα KO CD11c^+^ cells showed significantly increased binding to worms, which was abrogated when CD11a or Itgb7 was blocked (**Figure 4D**). These data indicate that RELMα KO CD11c^+^ cells gain an enhanced worm binding ability through increased expression of integrin alpha L, *Itgal*, and beta 7, *Itgb7*.

**Table 1:** NanoString advanced analysis of differentially expressed pathways in CD11c^+^-sorted cells from RELMα KO lungs compared with those from RELMα WT lungs at day 7 post *Nippostrongylus brasiliensis* infection.

Gene set analysis	Significance score	Down in KO	Up in KO
**Cell migration and adhesion**	17	*Retnla, Angpt2, Pf4, Cav1, Stab1, Itgam*	*Thbd, Itgb7, Amica1, Cldn1, Siglec1, Itgal, Cytip, H-dmb2, Ceacam1, Il17ra,F11r, Cd47*
**Complement activation**	4.8	*C3ar1, C1qb, C1qa, C1qc, Itgam*	*C5ar1, Thbd*
**TH1 activation**	3.3		*Il18, Il12b*
**Differentiation and maintenance of myeloid cells**	3.2	*Mafb*	*Marco, Top2a, Cytip, Mcm5*
**ECM remodeling**	2.9	*Mmp13, Adam8, Pdgfa, Itgam*	*Itgb7, Itgal, Ceacam1, F11r, Cd47*
**Th2 activation**	2.9		*Il6*
**TLR signaling**	2.7	*Tlr1, Ikbke, Ctsl, Itgam*	*Tlr3, Il12b, Il6, Il1b*
**Chemokine signaling**	2.7	*Pf4, Ccl12, Ccl9, Ccr5*	*Fpr1*
**Growth factor signaling**	2.7	*Flrt2, Amgpt2, Pdgfa, Jag1, Igf1, Hgf*	*Itgb7, Rictor, Il1a, Il12b, Fzd4, Il6, Klf10, Il1b*
**T-cell activation and checkpoint signaling**	2.6	*Tnfrsf14*	*Rictor*

### 3.5 Deletion of RELMα from the lung compartment contributes to transcriptional changes in repair-associated pathways at day 30 post infection

**Figure 5:**
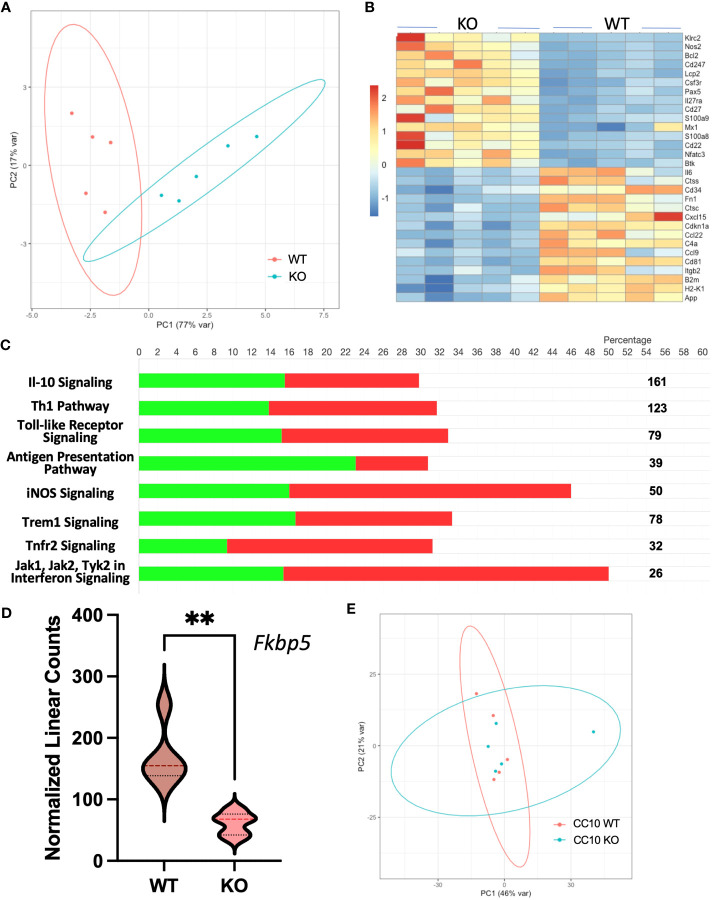
Gene expression analyses of lung cells from RELMα^ΔCC10^ and RELMα KO mice 30 days post infection reveal a role for RELMα in tissue repair. **(A)** Principal component analysis (PCA) plot depicting the clustering of RELMα KO versus WT samples based on NanoString data collected 30 days post *Nippostrongylus brasiliensis* infection. **(B)** Heatmap representation of differentially expressed genes (DEGs) in whole-lung digest cells isolated from infected RELMα KO and WT mice. **(C)** Ingenuity pathway analysis (IPA) illustrating the enrichment of functional pathways for (green, upregulated; red, downregulated number of genes) DEGs. **(D)** PCA plot depicting the clustering of CC10 RELMα^ΔCC10^ and RELMα^F/F^ samples based on NanoString gene data collected 30 days post *N. brasiliensis* infection. **(E)** Normalized linear count from CC10 NanoString data for *Fkbp5* (mean ± SEM; **p < 0.01).

To determine gene expression changes in the whole-lung compartment that may be associated with deficient tissue repair, gene expression analysis using the NanoString Mouse Immunology panel was performed on lung cell RNA from infected RELMα KO and RELMα^ΔCC10^ mice at day 30 post infection, along with their respective infected WT counterparts (RELMα WT and RELMα^F/F^). Principal component analysis (PCA) of RELMα WT versus RELMα KO indicated clustering by genotype (**Figure 5A**). There were 83 DEGs that met the cutoff criterion of *p*_adj_<0.05 and a heatmap of the top 30 DEGs was plotted **(** **Figure 5B** **)**. RELMα KO lung cells had an overrepresentation of genes involved in Th1-skewed or viral activation pathways such as the NK cell activation ligand *Klrc2*, *Nos2*, and the calcium-binding proteins *S100a8* and *S100a9*, which are biomarkers for inflammation. In contrast, WT lung cells expressed tissue repair-associated genes, such as *Fn1*, and chemokines. Ingenuity pathway analysis (IPA) of the DEGs between WT and RELMα KO lung cells indicated changes in JAK, cytokine, Tnfr2, antigen presentation, iNOS, toll-like receptors, and Th1 signaling pathways **(** **Figure 5C**; **Table 2** **).** When comparing gene expression in lung cells infected RELMα^ΔCC10^ and RELMα^F/F^ mice at 30 days post infection, PCA analysis did not reveal distinct clusters, indicating that CC10-specific deletion of RELMα was insufficient to alter overall lung cell gene expression **(** **Figure 5D** **)**. Indeed, only one gene was significantly downregulated in RELMα^ΔCC10^ lung cells, *Fkbp5*, which is involved in glucocorticoid activity **(** **Figure 5E** **)**. IPA analysis for global gene set patterns indicated alterations in wound healing, toll-like receptors, pathogen-induced cytokine storms, and iNOS signaling pathways (**Table 3** **)**. Pathways associated with airway inflammation in asthma were also affected. Many of these pathways were shared in the constitutive KO and WT lung cells, suggesting that the CC10-specific RELMα deletion had similar downstream effects as the constitutive deletion, but that the effects were more subtle. Overall, these transcriptional analyses indicate that constitutive but not CC10-specific RELMα deletion leads to the downregulation of genes associated with tissue remodeling and upregulation of inflammatory and Th1 cytokine activation pathways.

**Table 2:** Ingenuity Pathway Analysis of differentially expressed genes in lung cells from RELMα KO compared with those from RELMα WT mice at day 30 post *Nippostrongylus brasiliensis* infection.

Pathway	Down in KO	Up in KO
**Role of Jak1, Jak2, and Tyk2 in interferon signaling**	*Ifngr1*	*Relb*
**Tnfr2 signaling**	*Nfkbia, Tnfaip3*	*Relb*
**Interferon signaling**	*Ifngr1, Irf1*	*Bcl2, Tap1*
**Trem1 signaling**	*Il6, Il1b, Itgax, Mapk1, Tyrobp*	*Cd86, Irak1, Relb*
**iNOS signaling**	*Cd14, Ifngr1, Irf1, Mapk1, Nfkbia*	*Nos2, Relb, Irak1, Irak3*
**Antigen presentation pathway**	*B2 m, Cd74, Hla-a, Hla-Dqa1, Hla- d1b1*, *Hla-drb5, Tapbp*	*Hla-dob, tap1*
**Toll-like receptor signaling**	*Cd14, Il1a, Il1b, Il1rn*, *mapk1, nfkbia, tnfaip3*	*Irak1, Irak3, Relb*
**Th1 pathway**	*Hla-1, Hl1-dqa1, Hla-dqb1, Hl1-drb5, Ifngr1, Il6, Irf1, itgb2, Nfil3*	*Cd86, Cd247, Cd3d, Cd3e, Hla-dob, Il27ra, klrd1, nfatc3*
**Il10 Signaling**	*Cdkn1a, Hla-a, Hla-dob, Hla-dqa1*	*Bcl2, Cd86, Il1r1, Il1rap, nos2, Relb*

**Table 3:** Qiagen ingenuity pathway analysis of differentially expressed genes in lung cells from RELMα^ΔCC10^ compared with those from RELMα^F/F^ mice at day 30 post *Nippostrongylus brasiliensis* infection.

Pathway	Down in KO	Up in KO
**Wound healing signaling pathway**	*Il12b, Il18rap, Il1rap*, *Il1rl2*	
**Trem1 signaling**	*Il10, itga5*,	
**Pathogen-induced cytokine storm signaling pathway**	*Ccl2, Cxcl10, Cxcr3*, *Ifih1, Il10, Il12b, Il23a*	*Cxcr4, Hla-dmb*
**Il-10 signaling**	*Il10, Il1rap*,	*AHR, Hla-dmb*
**iNOS signaling**	*Irak1*	
**Toll-like receptor signaling**	*Il12b, Irak2*	
**Airway inflammation in asthma**	*Ccl20, Il-20, Il12b, Il23a*	

### 3.6 *Ex vivo* wound repair assay and acellular scaffolds indicate that helminth infection has persistent effects on the lung extracellular matrix

**Figure 6:**
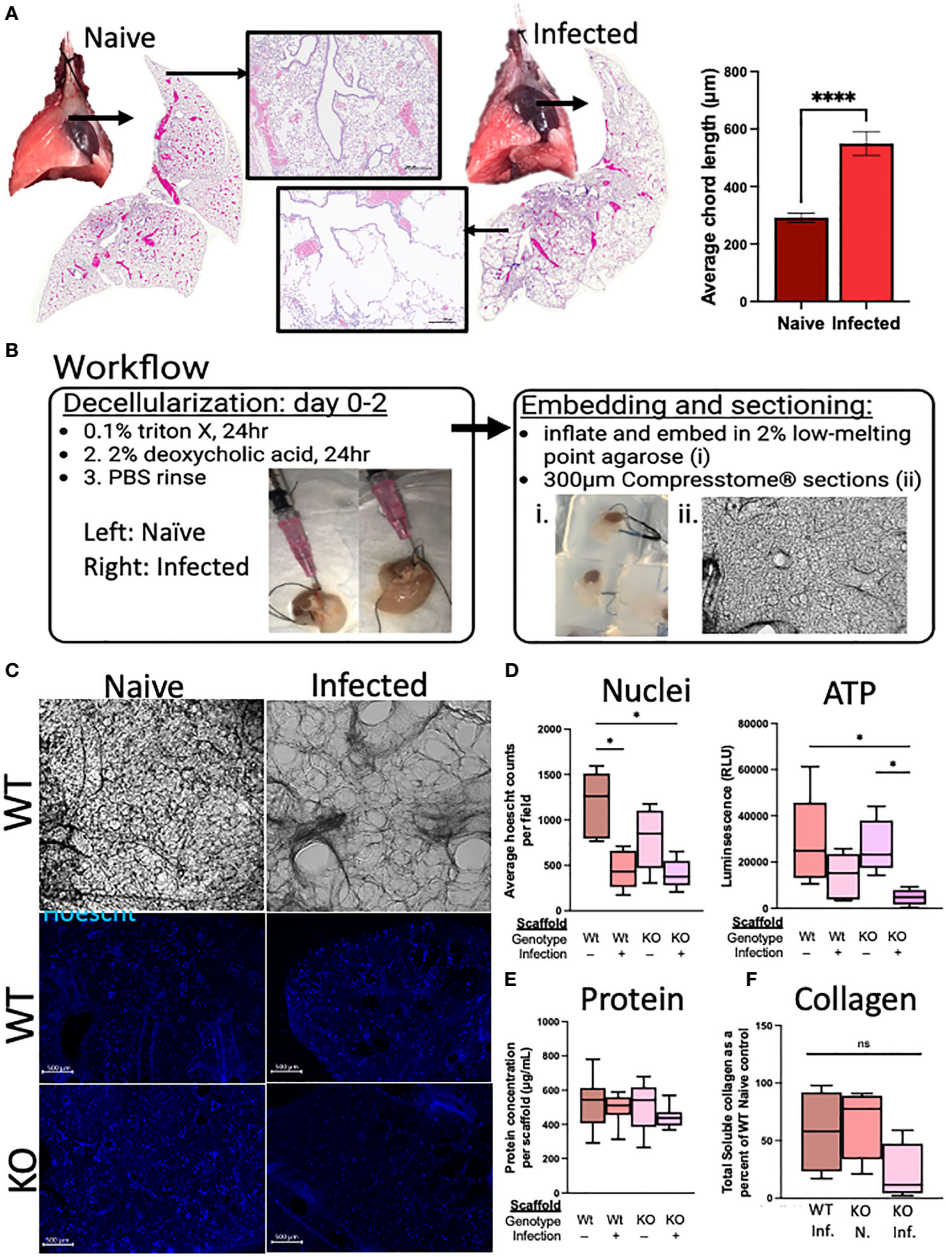
Deletion of RELMα has functional consequences for repair through epithelium–ECM interactions, as demonstrated using an *ex vivo* acellular lung scaffold repair assay. **(A)** Gross lung morphology is altered by infection owing to alveolar destruction (stitched image and 4× image). A quantifiable increase in chord lengths demonstrates an increased mean linear intercept as a measure of alveolar destruction (automated analysis package in ImageJ; *n* = 3 mice per group). **(B)** Workflow diagram for the lung decellularization process, agarose embedding, and sectioning of acellular scaffolds. Representative cellular scaffolds were Hoechst stained to confirm successful decellularization. **(C)** Representative images of scaffolds at day 6 of culturing with mouse epithelial cells (MLE12) after staining with Hoechst dye (4× obj., scale bar 500 microM). **(D)** Assessment of cellular activity is represented by nuclei counts (automated counting protocol ImageJ) and ATP assay (CellTiter-Glo 3D; Promega). **(E)** Protein was determined by a BCA assay from each individual scaffold, which was sonicated in 0.05% pepsin in acetic acid after cell lysis in the ATP assay. **(F)** Soluble collagen was determined from 100 mg of decellularized lung tissue (Abcam; *n* = 4). *Ex vivo* experiments were run five times with five biological replicates per group and three technical replicates per experiment. The averages were taken from each biological replicate for each experiment and plotted as box and whisker plots. *p < 0.05;****p < 0.0001, ns= not significant.

In the weeks following a single infection with *N. brasiliensis*, there are gross alterations to the lung appearance, with infected lungs marked by enlarged, nodular lobes even after the worms have left the lungs (**Figure 6A**). Stitched images demonstrate the diffuse alveolar destruction of H&E-stained FFPE lung sections. Using 20× images taken of the alveolar spaces, the mean linear intercept was determined by semi-automated quantification methods using an ImageJ plugin devised by Nolan et al (Crowley et al., 2019). An increasing chord length suggests further distances between tissue intersections, demonstrating a loss of alveolar surface area. These methods indicated that infected mice had significantly longer average chord lengths than naive mice, which suggests an emphysematous-like pathology post infection, as has been previously described by others (Marsland et al., 2008; Sutherland Id et al., 2018).

We sought to determine if RELMα deficiency during the infection recovery period affects the extracellular matrix (ECM), either directly through its role in collagen cross-linking or indirectly through RELMα-responsive cells with ECM remodeling roles, which could contribute to the lung repair process via epithelium–ECM interactions (Knipper et al., 2015). The decellularized lungs from RELMα WT and RELMα KO mice, both naive and infected, were inflated with agarose for vibratome sectioning (300-µm-thick sections) for *in vitro* re-epithelialization (**Figure 6B**). A mouse lung epithelial cell line (MLE-12-CRL2110) was plated onto each scaffold to assess the effectiveness of re-epithelialization over a period of 6 days. Hoechst staining of the scaffolds revealed epithelial integration onto each scaffold type, and alveolar destruction on the infected scaffolds was also apparent (**Figure 6C**). Nuclei counts were performed on 4× images of the scaffolds and only WT-infected mice (as opposed to WT-naive mice) showed significantly reduced average nuclei counts (**Figure 6D**). Cellular ATP, as an indicator of cell viability and metabolic activity, was measured and revealed a significant reduction in ATP from KO-infected scaffolds when compared with both WT and KO-naive scaffolds, suggesting that the epithelial cells on the scaffold from infected KO mice were less viable and/or less metabolically active. To normalize for potential differences in scaffold protein content between the groups, protein was extracted from each scaffold following lysis of cells during the ATP assay, which indicated that there were no significant differences in the protein content of the scaffolds used in these studies (**Figure 6E**). Acellular scaffolds were also assessed for soluble collagen content and no significant differences among the groups were observed (**Figure 6F**). Together, these acellular scaffold assays revealed that the lung architecture suffers from long-lasting changes in response to helminth infection, which negatively impacts subsequent re-epithelialization. The deficiency in RELMα did not significantly alter this deficit.

## 4 Discussion

Human soil-transmitted helminth infections and associated morbidities afflict billions of people each year; however, little is known of the long-term impacts of these infections on lung health. With staggering numbers of individuals affected among poor communities, it is imperative that we examine the potential impacts of these infections on the lungs, especially as lung diseases resulting from sustained lung damage, such as COPD, are highly prevalent in these regions (Brooker et al., 2006). Using the rodent parasite *N. brasiliensis*, which models these human infections, we sought to address the gaps in our knowledge surrounding the persisting pulmonary impacts of these infections.

Marsland et al. (2008), demonstrated progressive emphysematous-like pathologies in mice at 30–200 days post single infection with *N. brasiliensis*, indicating that even after these parasites have left the lungs their transient passage may have long-term consequences. The identification of protein regulators of this pathology to better understand this progression toward worsening emphysema would have implications for the treatment of emphysema and other pulmonary diseases resulting from alveolar damage. Here, we investigated the role of RELMα in emphysematous lung pathology as well as the cellular source of this persistent expression at 30 days post infection. We employed three transgenic mouse model systems to address myeloid- or airway epithelium-derived RELMα at this chronic time point. The use of these transgenic mouse models came with several caveats. In the tamoxifen-inducible CC10-Cre model, deletion of RELMα only occurred at the time of tamoxifen treatment and did not account for progenitor cells replenishing the CC10^+^ population, which could then express RELMα. In addition, CD11c^+^ subsets also include dendritic cells and some lymphocyte subsets, although past laboratory studies have shown that CD11c^+^ cells from the lungs are mainly macrophages based on morphology (Batugedara et al., 2018). Despite these caveats, these transgenic systems were validated: deletion of RELMα from CC10^+^ club cell progenitors was sufficient to significantly reduce RELMα concentration in BALF. This confirmed that epithelial cells are prominent secretors of RELMα in the airways. Although serum RELMα levels were unaffected in RELMα^ΔCC10^ mice, there was a significant reduction in serum RELMα in the RELMα^ΔCD11c^ animals. Thus, airway and blood RELMα derive from distinct cellular sources. As neither cell-specific knockout system fully depleted airway and serum RELMα, this highlights the redundancy in the expression of RELMα from varying cell-types, perhaps owing to the importance of its expression in mice recovering from helminth infection-induced lung injury.

To better understand the impact of elevated airway RELMα concentration on lung immune cell populations at the chronic recovery time point of day 30 post infection, we performed flow cytometry on cells isolated from BALF. We focused on distinguishing between tissue-resident (TR-) and monocyte-derived AMs, owing to their differing functions in lung repair and because monocyte-derived (Mo-) AMs secrete RELMα (Chen et al., 2022). Tissue-resident AMs play an important role in initiating tissue repair (Cheng et al., 2021), whereas monocyte-derived AMs have been linked to the pathogenesis of diseases such as fibrosis (McQuattie-Pimentel et al., 2018). Infection had a significant impact on the depletion of BAL AM subsets, even at 30 days post infection. Infection, but not RELMα deficiency, resulted in significantly decreased Mo-AM and TR-AM frequencies. Under homeostatic-naive conditions, BAL Mo-AM frequencies were affected by constitutive RELMα deletion, indicated by a deficit in mono-AM frequency in the airways of naive RELMα KO mice. In a recent publication by Sanin et al., using these same RELMα constitutive knockouts, it was found that cell-intrinsic expression of RELMα is critical in monocyte transition to macrophages (Sanin et al., 2022). Our data are supportive of these findings and suggest that monocytes rely on RELMα expression to facilitate their migration and inhabitation of tissues such as the lung.

The results from blinded histopathology scoring of lungs from 30 days post infection revealed significantly higher alveolar destruction scores in the infection group than in the naive controls. The cell-specific deletion of RELMα was insufficient to yield detectable differences in alveolar destruction by scoring. However, complete deletion of RELMα resulted in significantly higher alveolar destruction scores, underscoring the tissue protective role of RELMα during the lungs’ recovery from damage. These data support the assertion that RELMα plays a significant role in dampening the progressive alveolar destruction that follows helminth infection. The damage to the lungs during infection can arise from the parasites themselves as well as from the effects of excessive inflammation. To elucidate potential routes by which RELMα protects the lungs from worsening alveolar damage at day 30 post infection, we assessed an earlier time point and focused on the myeloid cells, which are responsible for interacting with the parasite and facilitating pathogen clearance. Gene expression analysis indicated that CD11c^+^ RELMα KO lung cells expressed genes associated with Th1 activation, inflammation, and cell migration, but were deficient in genes associated with wound healing. Furthermore, there was an increased level of expression of integrins, CD11a, and Itgb7, which were involved in the enhanced binding of RELMα KO cells to the *N. brasiliensis* parasites in the coculture. It is possible that this enhanced myeloid cell activation and interaction with the worm may cause lung damage and contribute to the latter lung pathology in the absence of RELMα, clarifying a myeloid-intrinsic role for RELMα. However, further studies would be needed to first address the parasite burden in the lung and intestine of the RELMα^ΔCD11c^ mice and, next, assess the levels of gene transcription of lung myeloid cells at the chronic time point of 30 days post infection. We have previously demonstrated that RELMα deficiency results in impaired complement pathway signaling, which was confirmed by our gene expression analyses in these studies. In mice completely deficient of RELMα and in RELMα KO CD11c^+^ cells and macrophages, there was a decreased level of expression of complement-related genes (Li et al., 2021). There is evidence here that RELMα may liaise with the complement system, suggesting a putative new role for this protein in modulating the innate immune response to pathogens and facilitating repair, particularly when myeloid cell populations are lacking RELMα (CD11c-specific KO and constitutive knockouts). These data underscore the importance of RELMα expression in facilitating an efficient immune response and initiating critical repair pathways, validated by the worsened alveolar destruction in the absence of RELMα seen in the histopathology in our studies. There is likely a time-dependent and cumulative effect of RELMα expression, which is why the phenotypes we observed were most apparent in the constitutive knockout animals, although there was sufficient overlap in the gene expression patterns and pathways across all the NanoString datasets. Specifically, genes associated with complement signaling, cell migration and adhesion, and innate immunity pathways were differentially expressed in the RELMα transgenic models investigated.

The long-term alteration in lung histology and structure in response to helminth infection can be seen in the significant increase in diffuse alveolar destruction. Gene expression analyses revealed deficits in tissue repair, cell proliferation, and ECM remodeling in the absence of RELMα. By modulating target cells, RELMα can impact extracellular matrix composition, and others have shown that RELMα can directly impact collagen cross-linking, which may be another mechanism by which RELMα facilitates lung repair in a cell-extrinsic manner (Knipper et al., 2015; Hauck et al., 2021). We investigated the possibility that RELMα may facilitate tissue repair via alterations to the ECM using *ex vivo* acellular lung scaffold repair assays. The structural damage to alveolar septa was visible on acellular scaffolds from infected mice, but all scaffolds had equivalent protein and collagen content and were able to support the growth of murine epithelial cell lines. Nonetheless, nuclei quantification indicated that the scaffolds from infected WT and RELMα KO mice supported significantly less epithelial cell growth than scaffolds isolated from naive WT mice. Metabolic activity was also assessed by ATP quantification, and cells grown on scaffolds from infected RELMα KO mice had significantly decreased levels of ATP compared with scaffolds from WT or KO-naive mice. These data suggest that RELMα may be altering the activity or proliferation of epithelial cells via its effect on the ECM. These data demonstrate an infection-dependent role of ECM–epithelium interactions in guiding repair, which may be influenced by RELMα. Ultimately, there are potential deficits in the initial attachment, maintenance, or proliferation of epithelial cells on ECM scaffolds from infected mice and those from a RELMα-deficient environment. This is an important and previously unexplored perspective in our understanding of how a single infection with helminths results in persistent alveolar damage and identifies specific proteins that may be involved, such as RELMα, which could be targeted for improved tissue repair. Other methods of protein analysis or stiffness assessment may reveal additional changes in the composition of the ECM that could explain how infection or RELMα changes the lung architecture and has a functional impact on epithelial cell growth and repair.

Overall, the implications of these data are that RELMα may play a critical role in initiating critical repairs in the lung following helminth infection. RELMα elicits these effects through cell-intrinsic effects as well as through extrinsic effects on the ECM. RELMα expression from either myeloid or epithelial sources was sufficient to elicit tissue-protective effects in this model; however, the cellular contributions of RELMα were compartment specific. Although the CC10-specific RELMα deletion was sufficient to significantly reduce BAL RELMα, the time points used in these studies did not allow for the complete deletion of RELMα from the airways. Furthermore, there are limitations in using CD11c-Cre as RELMα is not deleted from all myeloid cells. For future studies, the LysM-Cre mouse model may be more effective for the deletion of all myeloid subsets. It would also be valuable to identify the specific locations of RELMα expression in the context of lung injury using spatial transcriptomics, to clarify if RELMα is produced near the site of injury and repair and thus provide more context for RELMα expression and signaling. Ultimately, the investigation of RELMα signaling in lung repair could facilitate a better understanding of the fine line between successful or dysfunctional lung repair, which would have translational applications to human health and disease.

## Data availability statement

The gene expression data has been deposited in the NCBI repository, accession number(s) GSE236093; GSE236080; GSE236081.

## Ethics statement

The animal study was approved by University of California Riverside Institutional Animal Care and Use Committee. The study was conducted in accordance with the local legislation and institutional requirements.

## Author contributions

Experiment design: SS, SK, and TN. Day 30 experiments, tissue collection, flow cytometry, ELISA, and NanoString analysis: SS, SK, JJ, and SA. Histopathology: SS. Scaffold assays and analyses: SS and VB. Immunofluorescence analysis: SS and VB. CD11c day 7 NanoString analysis and coculture experiments: JL and JJ. Parasitology: KA and JJ. Data preparation: SS, SK, SA, and JJ. Manuscript preparation: SS and MN. Manuscript revision: JJ, SA, and MN. Project conceptualization: MN. All authors contributed to the article and approved the submitted version.
