# Supplementary material for: Myeloid- and epithelial-derived RELMα contribute to tissue repair following lung helminth infection
Source: Front Parasitol. Author manuscript; Available in PMC 2024 May 6. (PMC11073794; doi:10.3389/fpara.2023.1242866)
Supplement: Supplemental [file NIHMS1970684-supplement-Supplemental.pptx]

## Slide 1
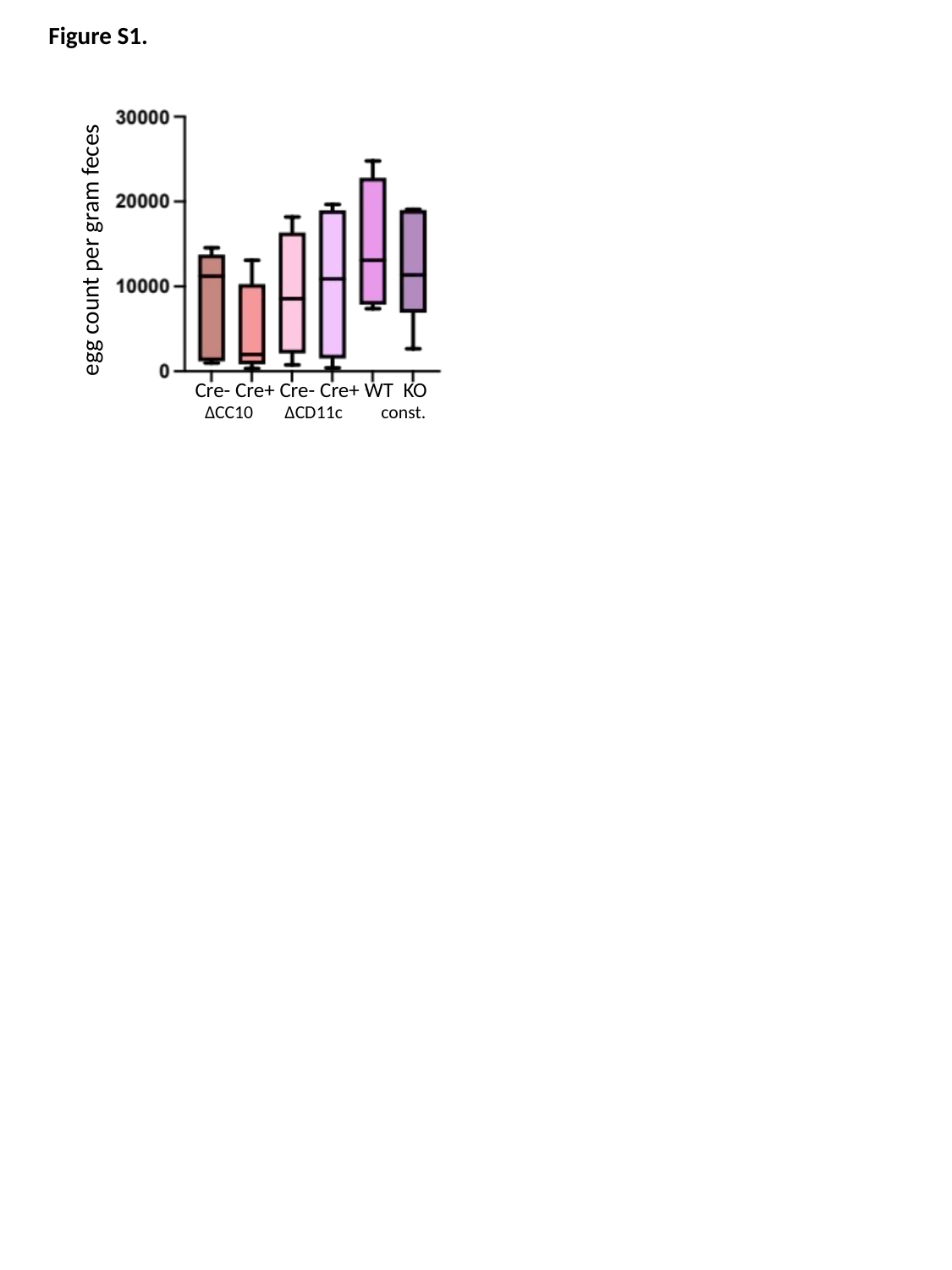

Figure S1.
egg count per gram feces
Cre- Cre+ Cre- Cre+ WT KO
ΔCC10
ΔCD11c
const.

## Slide 2
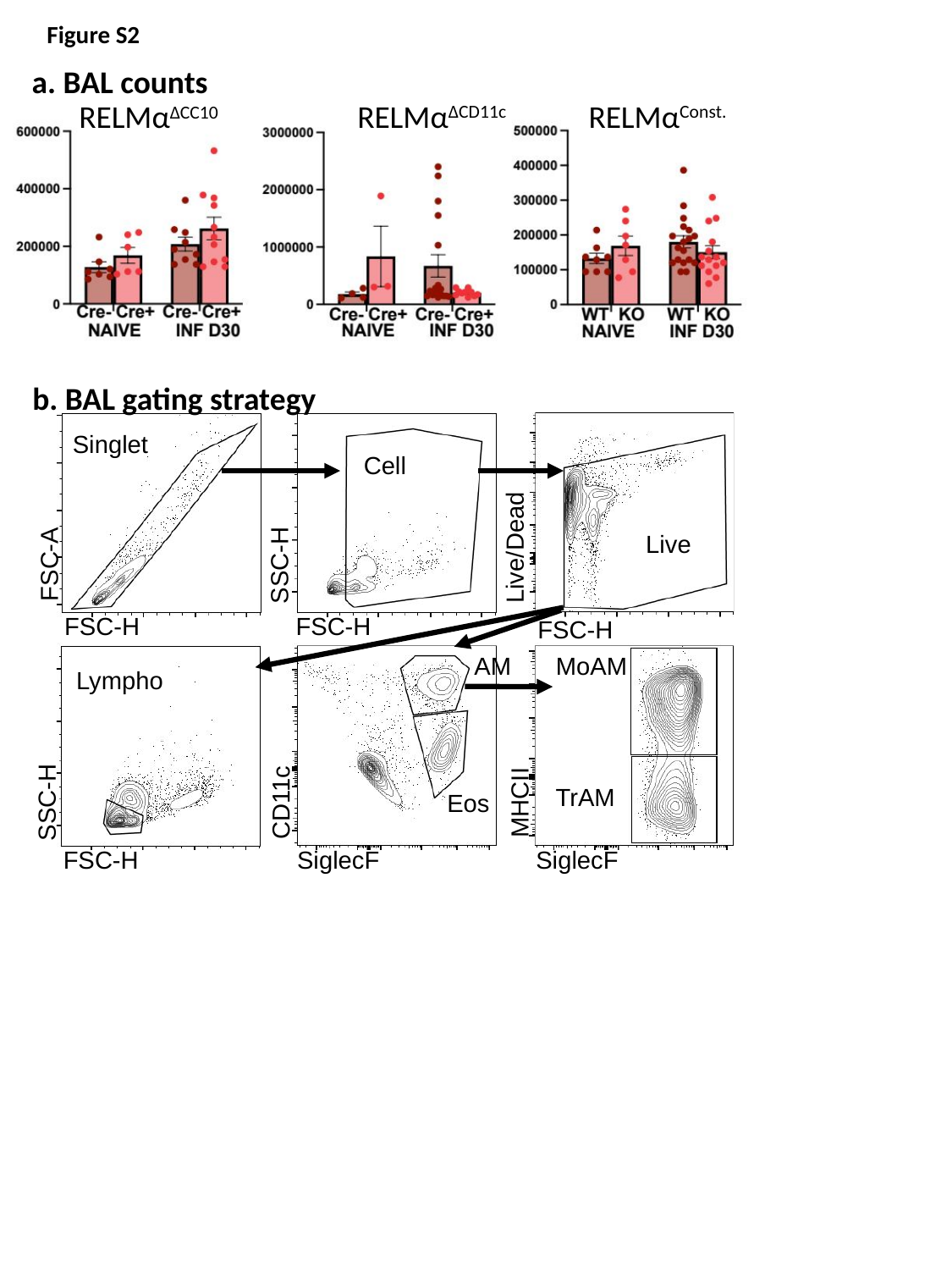

Figure S2
a. BAL counts
RELMαΔCD11c
RELMαConst.
RELMαΔCC10
b. BAL gating strategy
Singlet
Cell
Live
Live/Dead
FSC-A
SSC-H
FSC-H
FSC-H
FSC-H
MoAM
AM
Lympho
TrAM
SSC-H
MHCII
CD11c
Eos
FSC-H
SiglecF
SiglecF

## Slide 3
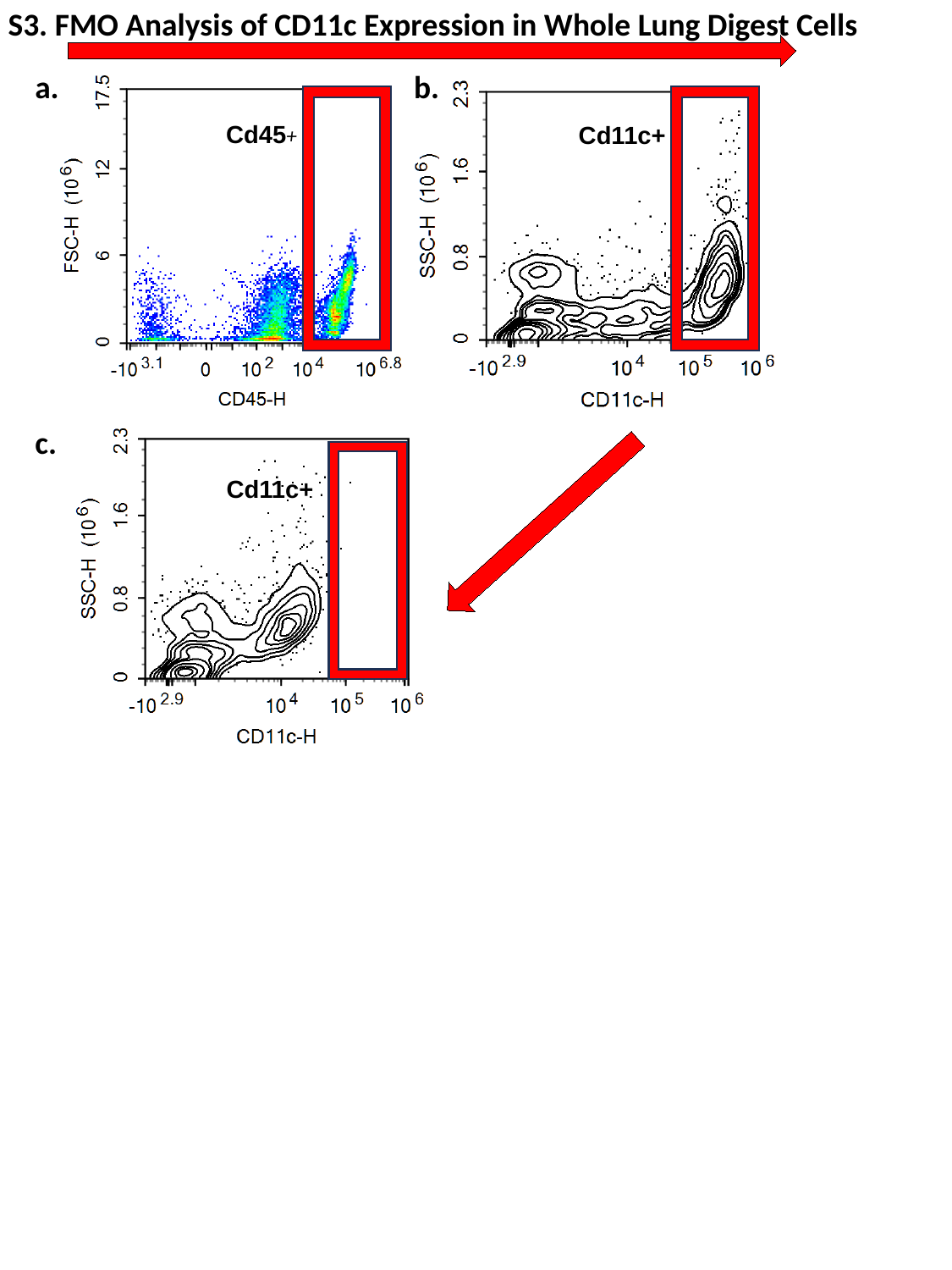

S3. FMO Analysis of CD11c Expression in Whole Lung Digest Cells
a.
b.
Cd45+
Cd11c+
c.
Cd11c+
